# Career construction model of adaptation: an empirical study among university students in Türkiye

**DOI:** 10.3389/fpsyg.2026.1865636

**Published:** 2026-10-05

**Authors:** Aşkay Nur Atasever, Metin Pişkin

**Affiliations:** 1 Department of Educational Sciences Guidance and Psychological Counseling, Faculty of Education, Afyon Kocatepe University, Afyonkarahisar, Türkiye; 2 Department of Guidance and Psychological Counseling, Faculty of Educational Sciences, Ankara Universitesi, Ankara, Türkiye

**Keywords:** academic satisfaction, career adaptability, career choice status, career construction model of adaptation, personal resources

## Abstract

Grounded in the Career Construction Model of Adaptation (CCMA), this study examined the direct and indirect associations among self-esteem, cognitive flexibility, general self-efficacy, career adaptability, career choice status (whether students chose their current department willingly), and academic satisfaction among university students in Türkiye. Particular attention was given to the joint associations of these personal resources with career adaptability and to career choice status as a potential link between adaptability and academic satisfaction. Cross-sectional data from 485 students at a public university were analyzed. An observed-variable structural equation model was estimated using unweighted least squares with 482 complete records, and uncertainty was assessed using 5,000 participant-level bootstrap resamples. Binary and ordinal logistic models were used as sensitivity analyses for the categorical outcomes. Self-esteem and cognitive flexibility were positively associated with general self-efficacy and career adaptability. The model accounted for 59.8% of the variance in career adaptability and 28.3% in academic satisfaction. Career adaptability was positively associated with career choice status, whereas the direct association between general self-efficacy and career choice status was not statistically significant. Both career adaptability and career choice status were positively associated with academic satisfaction. Bootstrap confidence intervals supported indirect associations of self-esteem and cognitive flexibility with career adaptability through general self-efficacy, of general self-efficacy with career choice status through career adaptability, and of career adaptability with academic satisfaction through career choice status. Sensitivity analyses showed consistent patterns for the corresponding direct associations. These findings provide evidence relevant to the CCMA by considering distinct personal resources and career choice status within an integrated model. However, the cross-sectional design and single-item measures of career choice status and academic satisfaction limit conclusions about developmental processes and causal mediation. The findings identify potential areas for further investigation and university career support.

## Introduction

The landscape of the contemporary labor market has undergone a paradigm shift, transitioning from the structured and predictable career paths of the 20th century to a highly volatile, uncertain, complex, and ambiguous (VUCA) environment in the 21st century. Driven primarily by rapid technological advancements and globalization, this transformation has been further exacerbated by macro-level disruptions such as global economic fluctuations, geopolitical conflicts, and public health crises (Savickas et al., 2009). Consequently, traditional employment frameworks have increasingly given way to non-standard, precarious work arrangements—including freelance, temporary, and gig economy roles—making occupational transitions more frequent and precarious (Koen et al., 2012; Nota et al., 2012). This destabilized ecosystem demands that upcoming entrants develop psychosocial resources and adaptive competencies that fundamentally diverge from the rigid skill sets required in the past era (Savickas et al., 2009).

This macroeconomic volatility directly reverberates into higher education, forcing university students to proactively navigate and construct their careers long before graduation (Akkermans et al., 2018). Beyond achieving academic proficiency, students must actively cultivate employability assets, engage in rigorous career exploration, and crystallize specific professional goals to facilitate a smooth school-to-work transition (Lent and Brown, 2013). Within the specific cultural and economic context of Türkiye, this transition is particularly fraught; empirical evidence indicates that Turkish university students experience severe career-related anxieties—such as post-graduation unemployment and inadequate career planning—which peak during their senior years (Atik and Yalçın, 2010; Karataş and Gizir, 2013). These apprehensions are further contextualized by national labor statistics, with the Turkish Statistical Institute (Turkish Statistical Institute, 2025) reporting a youth unemployment rate of 16.3% for 2024. Securing fulfilling employment stands as a definitive developmental task of young adulthood; hence, overcoming these structural barriers requires students to actively mobilize individual socio-cognitive and psychological resources (Hirschi, 2012). Foremost among these pivotal self-regulatory resources is career adaptability, a crucial construct embedded within the Career Construction Theory that enables individuals to cope with current and anticipated vocational transitions (Savickas et al., 2009).

Historically, the construct of career adaptability evolved from Super’s foundational concept of “career maturity” within his Life-Span, Life-Space theory. Super (1955, 1957) initially introduced career maturity to assess an adolescent’s readiness to cope with age-specific developmental tasks. However, as the contemporary labor market grew increasingly fluid, scholars recognized that maturity implied a static, predictable trajectory unsuitable for capturing lifelong development amid systemic shifts. To address this limitation, Super and Knasel (1981) proposed “career adaptability” to define an individual’s capacity to navigate changing work conditions. Savickas (1997) subsequently synthesized and broadened this construct as an individual’s readiness to cope with predictable or unpredictable vocational transitions and successfully adapt to emergent work roles, effectively superseding the traditional notion of career maturity across the life span (Niles and Bowlsbey, 2013).

Integrating this perspective into Career Construction Theory (CCT), Savickas (2002, 2005, 2013) framed the overall career development process as a dynamic, lifelong trajectory of self-regulatory adaptation. From this viewpoint, career adaptability represents a foundational psychological resource that enables individuals to actively construct, guide, and manage their occupational lives. Due to its robust explanatory power, career adaptability has emerged as one of the most widely researched constructs in vocational psychology and career counseling, prompting extensive meta-analytic and empirical investigations globally over the past two decades (Johnston, 2018; Rudolph et al., 2017a; Rudolph et al., 2017b). Parallel to this international body of research, evaluating the mechanism of career adaptability within the unique socioeconomic landscape of Türkiye is vital for understanding how university students systematically prepare for and execute their school-to-work transitions.

## Theoretical framework

### Career construction theory

Grounded in personal and social constructionism, Career Construction Theory (CCT; Savickas, 2002, 2005, 2013) represents a dynamic extension and contemporary reformulation of Super’s developmental framework. By integrating and updating the functional aspects of preexisting vocational paradigms, Savickas synthesized a comprehensive meta-theory that stands as a premier framework for explaining contemporary career development (Rudolph et al., 2019). CCT posits that individuals construct their careers by imposing meaning on their vocational behaviors and occupational transitions. To operationalize this interpretive process, the theory conceptualizes career construction through three distinct yet interrelated core dimensions: vocational personality, life themes, and career adaptability (Savickas, 2005).

While CCT incorporates vocational personality—including Holland’s RIASEC typology as discussed by Savickas (2005)—and life themes (Savickas, 2005, 2013) to capture interests and narrative career continuity, the operational core of the theory lies in its self-regulatory component. Neither rigid trait-matching nor purely descriptive narratives suffice to navigate the volatile 21st-century labor market. Instead, individuals must actively synthesize the self with changing environments through a transactional, psychosocial process (Savickas, 2005).

Consequently, career adaptability emerges as the functional engine of CCT, denoting the psychosocial resources, competencies, and self-regulatory behaviors that individuals deploy to fit themselves into congruent work environments. Cultivating career adaptability is essential for modern workforce entrants, as it equips individuals to master developmental tasks, navigate unpredictable vocational transitions, and effectively cope with occupational traumas or unexpected market shifts (Savickas, 2013).

### The career construction model of adaptation (CCMA)

From an empirical standpoint, the ultimate objective of career interventions within CCT is to empower individuals to self-regulate and manage their occupational trajectories effectively. To operationalize the psychological mechanisms underlying this capacity, Savickas (2013) proposed the Career Construction Model of Adaptation (CCMA). This conceptual model delineates a rigorous, hierarchical sequence that maps the transition from adaptive readiness to ultimate psychological outcomes (see Figure 1). The sequential framework consists of four progressive stages, visually conceptualized as follows.

**Figure 1:**
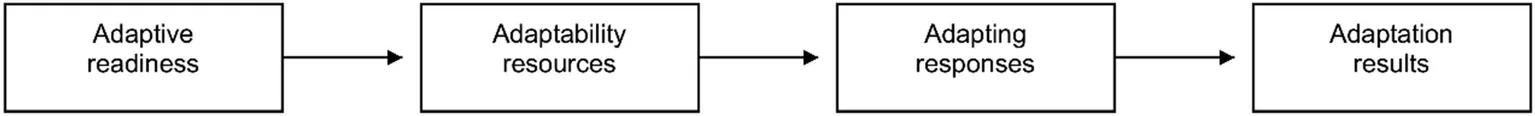
The career construction model of adaptation. The diagram summarizes the proposed sequence from adaptive readiness through adaptability resources and adapting responses to adaptation results. Redrawn based on Savickas and Porfeli (2012) and Rudolph et al. (2017b).

#### Components of the model

##### Adaptive readiness (adaptivity)

According to Career Construction Theory (CCT), adaptive readiness (or adaptivity) signifies an individual’s psychological flexibility, willingness, and trait-like readiness to respond effectively to developmental career tasks, occupational transitions, and vocational traumas (Savickas, 2013). Savickas conceptualizes adaptivity as a relatively stable psychological disposition that enables individuals to sustain goal-directed personal and interpersonal actions aimed at self-and-environmental modification during periods of vocational instability. To successfully adapt to systemic shifts, individuals must first possess the underlying capacity for self-regulation. Consequently, adaptive readiness serves as the essential psychological bedrock that fosters the development and mobilization of specific coping mechanisms, termed adaptability resources. This internal readiness can be operationalized through core psychological traits, such as cognitive flexibility, self-esteem, and general self-efficacy. Empirically, the activation of adaptability resources is driven by individual factors—including cognitive ability, optimism, proactive personality, and hope (Johnston, 2018; Rudolph et al., 2017a; Rudolph et al., 2017b; Savickas and Porfeli, 2012)—as well as contextual assets, such as transformational leadership (Lan and Chen, 2020), supportive parenting (Soresi et al., 2014), and broader socio-environmental support systems (Ataç et al., 2018; Duffy, 2010; Karacan-Özdemir, 2016; Tian and Fan, 2014).

##### Adaptability resources (adaptability)

Career adaptability represents a psychosocial construct comprising the self-regulatory capacities and resources that facilitate an individual’s ability to cope with current or anticipated vocational tasks, professional transitions, and work-related stressors (Savickas, 1997, 2013; Savickas and Porfeli, 2012). Rather than representing static traits, they function as dynamic self-regulatory powers that shape the specific strategies individuals deploy to solve complex, ill-defined, and unexpected occupational problems, collectively operationalized through four interrelated dimensions (Savickas and Porfeli, 2012).

Career Concern constitutes the foundational dimension, representing planful anticipation and awareness of one’s future professional roles, where a constructive level of professional vigilance acts as a motivating catalyst for proactive career preparation (Öncel, 2014).

Career Control denotes the self-regulatory responsibility individuals assume for their professional futures, empowering independent decision-making and self-discipline to take ownership of their occupational trajectories.

Career Curiosity drives the proactive exploration of possible future selves and alternative occupational environments to evaluate the person-environment fit between emerging vocational identities and the labor market.

Career Confidence reflects an individual’s self-efficacy beliefs regarding their capacity to overcome obstacles and actualize their professional goals, systematically increasing the probability of attaining subjective and objective career success (Savickas, 2002, 2013).

##### Adapting responses (adapting)

Occupying the third tier of the CCMA, adapting responses encompass the actual behaviors, beliefs, and strategic actions that individuals manifest to navigate developmental tasks and fluid market conditions (Hirschi et al., 2015; Rudolph et al., 2017a; Savickas, 2013). Within the model’s sequential hierarchy, underlying adaptability resources directly fuel these adapting responses. Individuals who successfully mobilize their 4C resources are better equipped to resolve vocational problems, steer their career paths intentionally, and exhibit resilience against environmental adversity. Extant literature confirms that career adaptability resources robustly predict adaptive behaviors and agency, such as systematic career planning, diminished career decision-making difficulties, intensive career exploration, and heightened occupational, entrepreneurial, or career decision-making self-efficacy (Hirschi et al., 2015; Johnston, 2018; Rudolph et al., 2017a; Rudolph et al., 2019; Savickas and Porfeli, 2012). These concrete actions signify that latent adaptability resources are being actively operationalized into manifest career behaviors.

##### Adaptation outcomes (adapted)

The ultimate functional goal of the career construction process is to achieve a sustainable person-environment alignment between an individual’s personal values and the fluctuating demands of the occupational sphere. Adaptation outcomes represent the final, evaluative gains achieved at the end of this sequential self-regulatory chain. These outcomes are typically manifested through psychological indices of vocational success, systemic development, enduring well-being, and occupational integration (Rudolph et al., 2017a; Rudolph et al., 2017b; Savickas and Porfeli, 2012). Empirical frameworks demonstrate that career adaptability is robustly tied to adaptive outcomes such as enhanced employability, institutional promotability, elevated job, school, and career satisfaction, and organizational commitment. Conversely, it serves as a powerful protective factor against maladaptive outcomes, including turnover intentions and chronic job stress (Johnston, 2018; Rudolph et al., 2017a; Rudolph et al., 2017b).

## The current study

Grounded in the Career Construction Model of Adaptation (CCMA; Savickas, 2013), the current study examines a theory-specified model of direct and indirect associations in the higher education context in Türkiye. Specifically, self-esteem, cognitive flexibility, and general self-efficacy represent personal characteristics relevant to adaptive readiness; career adaptability represents adaptability resources; career choice status provides a limited, retrospective indicator relevant to adapting responses; and academic satisfaction represents an adaptation outcome. Examining these relationships within the same model allows the distinct associations of the personal characteristics with career adaptability, and the proposed indirect associations involving career choice status and academic satisfaction, to be assessed (see Figure 2).

**Figure 2:**
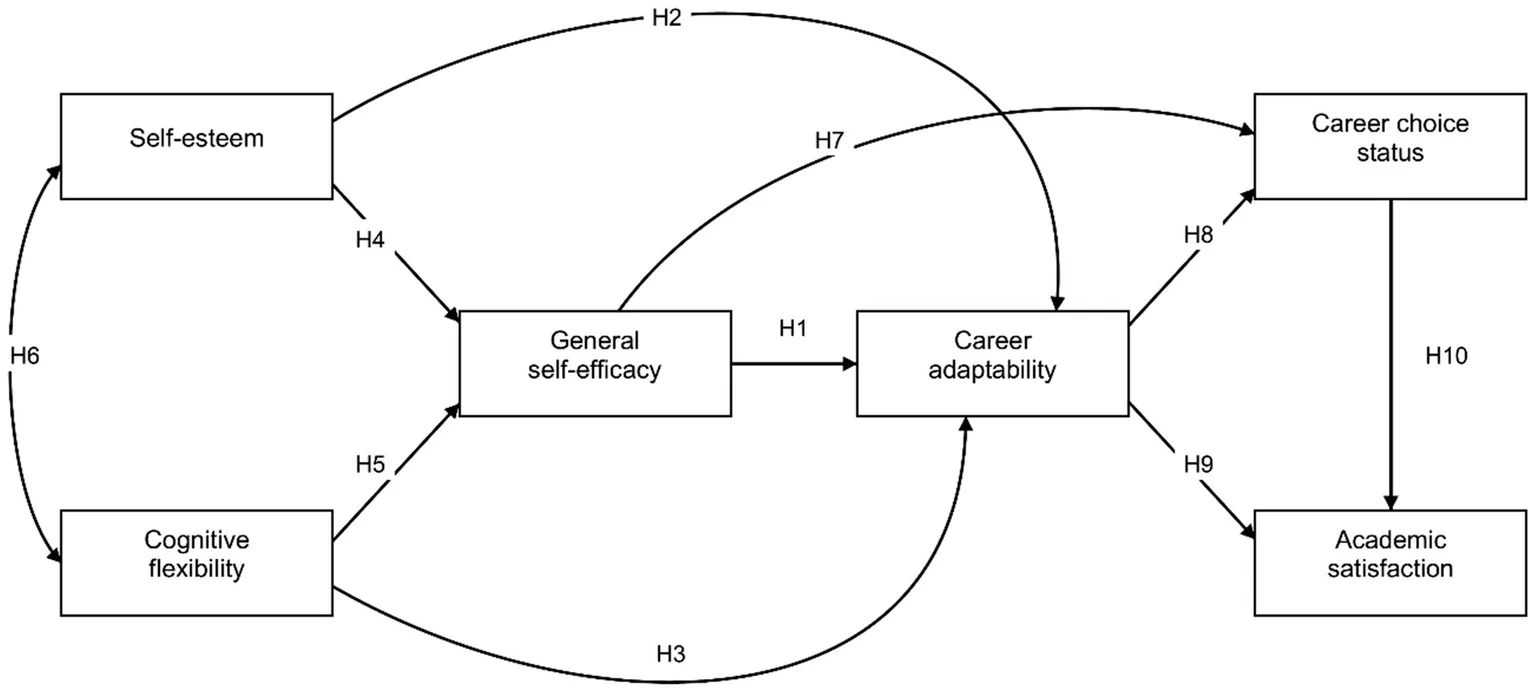
The hypothesized structural path model. Single-headed arrows indicate the specified directional paths (H1–H5 and H7–H10). The double-headed arrow represents the covariance between self-esteem and cognitive flexibility (H6). H11–H14 concern products of the relevant paths and are reported in Table 5. Residual terms are omitted for clarity. The specified directions do not establish temporal ordering in the cross-sectional data.

### Hypotheses development and rationale

#### Adaptive readiness precursors and career adaptability

The first stage of the proposed structural model examines the joint associations of self-esteem, general self-efficacy, and cognitive flexibility with career adaptability, treating these personal characteristics as indicators relevant to adaptive readiness. Self-esteem, defined as an individual’s subjective evaluation of their intrinsic worth, capability, and competence (Coopersmith, 1967; Rosenberg, 1965), serves as a critical self-regulatory anchor. Extant literature establishes that while low self-esteem predicts prolonged unemployment (Huysse-Gaytandjieva et al., 2015), elevated self-esteem intensifies proactive job search (Kanfer et al., 2001), enhances employability (McArdle et al., 2007), and is associated with life satisfaction (Lee et al., 2021; Marcionetti and Rossier, 2021). Parallel to this, general self-efficacy—the enduring belief in one’s capacity to cope with cross-situational stressors and demanding life tasks (Luszczynska et al., 2005)—equips workforce entrants with the psychological resilience needed to navigate institutional transitions. Empirical evidence confirms that general self-efficacy robustly triggers adaptive career behaviors, including comprehensive career planning, intensive environmental exploration, and entrepreneurial intentions (Atitsogbe et al., 2019; Van der Horst et al., 2017; Xu, 2020).

Furthermore, cognitive flexibility—characterized by an awareness of multifaceted behavioral options, adaptability to novel stimuli, and a sense of situational competence (Martin and Anderson, 1998; Martin and Rubin, 1995)—aligns perfectly with the core tenets of Career Construction Theory (CCT). Savickas (1997, 2013) posited that successfully navigating a turbulent, non-linear labor market requires an inherent willingness to alter personal behaviors to fit emergent environmental demands. Consequently, cognitive flexibility serves as an operational prerequisite for developing the “4Cs” of career adaptability. While prior research has documented positive associations of self-esteem (Ataç et al., 2018; Cai et al., 2015; Öztemel and Yıldız-Akyol, 2021), general self-efficacy (Karacan-Özdemir, 2016; Marcionetti and Rossier, 2021; Xu, 2020), and cognitive flexibility (Chong and Leong, 2017; Yıldız-Akyol and Boyacı, 2020) with career adaptability, the present study examines their simultaneous contribution within a unified CCMA framework. Examining these precursors concurrently provides a more holistic understanding of how internal adaptive readiness translates into manifest adaptive resources. More specifically, including these variables in the same model allows their distinct associations with career adaptability to be assessed while accounting for their interrelationships, including the proposed indirect associations of self-esteem and cognitive flexibility with career adaptability through general self-efficacy. Based on these theoretical underpinnings, we propose the following hypotheses:

*H1*: General self-efficacy is directly and positively related to career adaptability.

*H2*: Self-esteem is directly and positively related to career adaptability.

*H3*: Cognitive flexibility is directly and positively related to career adaptability.

*H4*: Self-esteem is directly and positively related to general self-efficacy.

*H5*: Cognitive flexibility is directly and positively related to general self-efficacy.

*H6*: There is a positive association between self-esteem and cognitive flexibility.

*H11*: Self-esteem is positively and indirectly associated with career adaptability through general self-efficacy.

*H12*: Cognitive flexibility is positively and indirectly associated with career adaptability through general self-efficacy.

#### Career adaptability and career choice status

Vocation stands as a primary structural pillar that shapes individual life meaning and longitudinal life satisfaction (Yeşilyaprak, 2019). Consequently, executing an autonomous, intrinsic career choice during emerging adulthood is a critical developmental milestone. Within the institutional context of Türkiye, this process is uniquely constrained; higher education entry is determined by a highly competitive, centralized university placement examination (Korkut-Owen et al., 2012; Pişkin, 2012). This structural rigidity often forces students into academic tracks based on test scores rather than vocational interests. Given this backdrop, evaluating a student’s career choice status—operationalized as whether they reported choosing their current academic department willingly—becomes particularly relevant. This indicator captures a specific aspect of students’ educational choices rather than the full range of adapting responses.

According to the CCMA, mobilizing adaptability resources (Concern, Control, Curiosity, and Confidence) directly shapes adapting responses, such as enrollment choices. Specifically, students must anticipate their future roles (concern), take autonomous ownership of their decisions (control), explore person-environment compatibility (curiosity), and maintain the conviction to execute their goals (confidence). Individuals with robust career adaptability resources are therefore better equipped to achieve alignment between their vocational self-concepts and their academic majors, a linkage confirmed by emergent empirical data (Erus and Zeren, 2017; Perera and McIlveen, 2017; Yıldız-Akyol and Boyacı, 2020). Conversely, students mismatched with their academic tracks exhibit high rates of departmental migration (Gizir, 2005; Kavas, 2005) or institutional attrition (Bülbül, 2012; Uslu, 2017), resulting in substantial psychosocial and economic losses. Thus, career adaptability is expected to be positively associated with students’ reports of having chosen their academic department willingly. Therefore, we hypothesize:

*H7*: General self-efficacy is directly and positively related to career choice status.

*H8*: Career adaptability is directly and positively related to career choice status.

*H13*: General self-efficacy is positively and indirectly associated with career choice status through career adaptability.

#### Career adaptability, career choice status, and academic satisfaction

The ultimate evaluative endpoint within the current CCMA framework is academic satisfaction, conceptualized as a positive emotional state stemming from the subjective fulfillment of an individual’s educational expectations and major-specific choices (Urbanaviciute et al., 2016). Drawing strong theoretical parallels to job satisfaction, the academic environment functions as a preliminary occupational theater where students operationalize their evolving self-concepts, skills, and interests (Allen, 1996; Jadidian and Duffy, 2012). Academic satisfaction yields critical educational dividends, including superior performance, sustained engagement (Nauta, 2007; Wilkins et al., 2014), and positive spillover into subsequent workplace satisfaction.

Extant longitudinal and cross-sectional research provides evidence of associations between career adaptability and academic satisfaction (Celik and Storme, 2018; Duffy et al., 2015; Nilforooshan, 2020; Urbanaviciute et al., 2016; Xu, 2020). Related research has also linked career optimism to academic major satisfaction and career choice satisfaction (McIlveen and Perera, 2016). The current study extends this line of inquiry by examining career choice status as a specific indicator relevant to adapting responses and as a potential link between career adaptability and academic satisfaction. This approach allows the proposed indirect association between adaptability resources and adaptation outcomes to be examined within the Turkish higher education context, without assuming that cross-sectional associations establish a temporal or causal sequence. Consequently, the following hypotheses are formulated:

*H9*: Career adaptability is directly and positively related to academic satisfaction.

*H10*: Career choice status is directly and positively related to academic satisfaction.

*H14*: Career adaptability is positively and indirectly associated with academic satisfaction through career choice status.

## Method

### Research design

This study used a cross-sectional correlational design to examine the direct and indirect associations proposed by the Career Construction Model of Adaptation (CCMA) among university students. The hypothesized relationships among personal resources, career adaptability, career choice status, and academic satisfaction were specified as an observed-variable path model within the structural equation modeling (SEM) framework. Although the direction of the hypothesized paths was guided by theory, the cross-sectional design does not establish temporal precedence or causality.

### Participants and data screening

Participants were recruited through convenience sampling from five faculties at Afyon Kocatepe University in Türkiye: Education, Science and Letters, Economics and Administrative Sciences, Engineering, and Veterinary Medicine. Data were collected during the spring semester of the 2018–2019 academic year. Of the 510 questionnaires collected, 25 were excluded because of incomplete responses, leaving a dataset of 485 participants.

The sample included 103 students from Education (21.2%), 100 from Science and Letters (20.6%), 98 from Economics and Administrative Sciences (20.2%), 99 from Engineering (20.4%), and 85 from Veterinary Medicine (17.5%). Participants were enrolled in their first (*n* = 100), second (*n* = 76), third (*n* = 179), or fourth (*n* = 130) year of study. Gender was recorded as female for 294 participants (60.6%) and male for 190 participants (39.2%); one record contained an undefined gender code (0.2%).

Three responses to the career choice status item contained codes outside the defined response categories and were treated as missing. Among the 482 valid responses, 362 participants (75.1%) reported choosing their current department willingly, whereas 120 (24.9%) reported not doing so. Responses to the four multi-item measures and the academic satisfaction item were available for all 485 participants.

Regarding academic satisfaction, 243 participants reported being satisfied and 98 completely satisfied with their department, together accounting for 70.3% of the sample. A further 92 participants (19.0%) selected the midpoint response, while 39 reported being dissatisfied and 13 not at all satisfied, together accounting for 10.7%.

Sample size was considered in relation to general SEM guidance and the complexity of the proposed structural model. Kline (2016) identifies approximately 200 cases as a typical sample size in SEM research, while emphasizing that requirements vary with model complexity, distributional characteristics, and the estimation method. The structural model was estimated using 482 complete records and comprised six observed variables and 16 free parameters, corresponding to approximately 30 observations per estimated parameter. This provided a practical sample-size rationale for the relatively parsimonious model, although the ratio was not treated as a formal adequacy criterion for unweighted least squares estimation with nonnormally distributed variables.

### Data collection instruments

The complete Turkish item wording, response options, and scoring instructions for the four multi-item measures and the two single-item indicators, including reverse-scoring information where applicable, are provided in Supplementary material S2.

#### Personal information form

A researcher-developed personal information form collected information on participants’ gender, age, faculty, department, and year of study. It also included two single-item indicators used to represent adapting responses and adaptation outcomes within the proposed CCMA framework.

*Career choice status*: Participants answered the question, “Did you choose your current department willingly?” Valid responses were coded as 1 (yes) and 0 (no). This item assessed participants’ retrospective reports of whether their department choice was voluntary. It was used as a limited indicator of an adapting response rather than as a comprehensive measure of career exploration, decision-making, or autonomous motivation. Its categorical format directly captured the distinction of interest, although a single item cannot represent the full range of adapting responses specified by the CCMA.

*Academic satisfaction*: Satisfaction with the current academic department was assessed using a single item rated from 1 (not at all satisfied) to 5 (completely satisfied). Higher scores indicated greater satisfaction. The item provided a global evaluation of a clearly specified target—the participant’s current department—rather than a multidimensional assessment of academic adjustment or university life.

These single-item indicators were used to address narrowly defined aspects of department choice and satisfaction. Nevertheless, their limited content coverage and potential measurement error should be considered when interpreting the findings. Internal consistency coefficients cannot be calculated for single-item measures.

### Career adapt-abilities scale

Career adaptability was assessed using the Career Adapt-Abilities Scale (CAAS; Savickas and Porfeli, 2012), adapted into Turkish by Buyukgoze-Kavas (2014). The scale contains 24 items distributed equally across four dimensions: concern, control, curiosity, and confidence. Participants rated each item on a five-point scale ranging from 1 (not strong at all) to 5 (very strong). The total score ranges from 24 to 120, with higher scores indicating greater career adaptability. The total score was used as the observed indicator of adaptability resources in the structural model.

### General self-efficacy scale

General self-efficacy was measured using the General Self-Efficacy Scale (Schwarzer and Jerusalem, 1995), adapted into Turkish by Aypay (2010). The scale comprises 10 items rated from 1 (completely false) to 4 (completely true). Item scores were summed to obtain a total score ranging from 10 to 40. Higher scores indicate stronger beliefs in one’s capacity to cope with difficult or demanding situations. The total score was used in the structural model.

### Rosenberg self-esteem scale

Global self-esteem was assessed using the 10-item self-esteem measure developed by Rosenberg (1965) and adapted into Turkish by Çuhadaroğlu (1986). In this study, responses ranged from 1 (strongly disagree) to 4 (strongly agree). The five negatively worded items—Items 3, 5, 8, 9, and 10 in the administered version—were reverse-scored. The 10 item scores were summed, yielding a total score ranging from 10 to 40, with higher scores indicating greater self-esteem. This total score was used in the structural model.

### Cognitive flexibility inventory

Cognitive flexibility was assessed using the Cognitive Flexibility Inventory (Dennis and Vander Wal, 2010), adapted into Turkish by Sapmaz and Doğan (2013). The inventory contains 20 items representing two dimensions: Alternatives, comprising 13 items assessing the ability to consider alternative explanations and solutions, and Control, comprising seven items assessing the tendency to perceive difficult situations as controllable.

In the Turkish version administered in this study, responses ranged from 1 (not at all appropriate) to 5 (completely appropriate). Items 2, 4, 7, 9, 11, and 17 were reverse-scored. The total score ranges from 20 to 100, with higher scores indicating greater cognitive flexibility. Although the inventory provides subscale scores, its total score was used as the observed cognitive flexibility variable in the structural model.

### Data analysis

#### Preliminary analyses

Data screening and descriptive analyses were conducted using IBM SPSS Statistics, version 25. Frequencies and percentages were calculated for categorical variables, and means and standard deviations were calculated for scale scores. Response codes were checked against the defined response categories, and undefined codes were treated as missing.

Analyses of the four multi-item measures used all 485 participants, whereas structural analyses used the 482 records with complete data on all model variables.

Distributional characteristics were examined using skewness and kurtosis statistics and a multivariate normality assessment. Potential multicollinearity was examined using correlations, variance inflation factors, and tolerance statistics. Bivariate associations among the four multi-item scale scores were summarized using Pearson correlations. Associations between career choice status and these scale scores were examined using point-biserial correlations. All associations involving academic satisfaction, including its association with career choice status, were examined using Spearman rank correlations. The structural model was estimated from a separate Pearson correlation matrix, as described below.

Internal consistency was assessed using Cronbach’s alpha and model-based omega total for the four multi-item scale scores. Separate confirmatory factor analyses were conducted using semopy, version 2.3.11, with ULS estimation applied to Pearson item correlation matrices. The specified structures comprised four correlated factors for career adaptability, two correlated factors for cognitive flexibility, and one factor each for self-esteem and general self-efficacy. These analyses used all 485 participants and treated item responses as numeric scores. Standardized factor loadings and SRMR were examined. Omega total was calculated as the model-implied common-factor variance of each total score divided by its model-implied total variance. For multidimensional measures, this coefficient included common variance across the correlated factors and was not interpreted as evidence of unidimensionality.

#### Structural equation modeling

The hypothesized relationships were examined using observed-variable path analysis within the structural equation modeling framework. The model was estimated using semopy, version 2.3.11. Self-esteem and cognitive flexibility were specified as correlated exogenous variables. General self-efficacy was regressed on self-esteem and cognitive flexibility, and career adaptability was regressed on general self-efficacy, self-esteem, and cognitive flexibility. Career choice status was regressed on general self-efficacy and career adaptability. Academic satisfaction was regressed on career adaptability and career choice status.

Parameters were estimated jointly using unweighted least squares (ULS) applied to the Pearson correlation matrix of the six study variables. Analysis of the correlation matrix placed the variables on a common scale for the ULS fitting criterion. Career choice status was coded as 0 (involuntary enrollment) or 1 (voluntary enrollment), and academic satisfaction was represented by its observed score from 1 to 5. The analysis therefore used a linear observed-score model rather than a categorical latent-response model. Sensitivity analyses using categorical outcome models were conducted to examine this modeling choice.

The model contained nine directional paths, one covariance between the exogenous variables, and six variance parameters, yielding 16 free parameters and five degrees of freedom. Overall model fit was evaluated using the standardized root mean square residual (SRMR) and Browne’s (1984) residual-based asymptotically distribution-free test. The latter was calculated in Python from the fitted model and an empirical fourth-moment estimate of the asymptotic covariance matrix of the sample covariance moments. The test statistic was evaluated against a chi-square distribution with five degrees of freedom. Explained variance was calculated for each endogenous variable as one minus the ratio of its estimated residual variance to its model-implied variance.

#### Bootstrap inference and indirect associations

Sampling uncertainty was assessed using 5,000 participant-level nonparametric bootstrap resamples drawn with replacement from the 482 complete records. The correlation matrix and the full structural model were re-estimated in each resample. All 5,000 bootstrap solutions converged.

Unstandardized coefficients were expressed in the original score units, and standardized coefficients were calculated using model-implied variances. Bootstrap standard errors and 95% percentile confidence intervals were calculated for direct and specific indirect associations. Approximate two-sided Wald *p* values for direct paths were obtained by dividing the unstandardized coefficients by their bootstrap standard errors and referring the resulting statistics to the standard normal distribution. Inference for indirect associations was based on their percentile confidence intervals.

Four specific indirect associations were calculated as products of the relevant path coefficients: self-esteem with career adaptability through general self-efficacy; cognitive flexibility with career adaptability through general self-efficacy; general self-efficacy with career choice status through career adaptability; and career adaptability with academic satisfaction through career choice status. A 95% confidence interval excluding zero was interpreted as evidence of a nonzero indirect association.

The direction of the paths was informed by the CCMA. Given the cross-sectional design, the estimated indirect associations were not interpreted as evidence of temporal ordering or causal mediation. A nonsignificant direct association was not considered sufficient evidence of complete mediation. Bootstrap resampling treated participant records as independent and did not account for potential classroom clustering.

#### Sensitivity analyses

Sensitivity analyses examined the direct associations involving the binary and ordinal outcomes. Career choice status was modeled using binary logistic regression with general self-efficacy and career adaptability as predictors and HC3 heteroskedasticity-robust standard errors. Academic satisfaction was modeled using ordinal logistic regression with career adaptability and career choice status as predictors. The ordinal model assumed proportional odds.

These analyses assessed whether the direction and statistical evidence for the corresponding direct associations were maintained when categorical outcome models were used. Their coefficients were not treated as equivalent to the linear SEM coefficients and were not used to calculate the SEM indirect associations.

## Findings

### Descriptive statistics and bivariate associations

Descriptive statistics are presented in Table 1. Among the 485 participants, mean scores were 91.50 (SD = 15.67) for career adaptability, 76.11 (SD = 11.21) for cognitive flexibility, 31.67 (SD = 5.30) for self-esteem, and 31.81 (SD = 5.48) for general self-efficacy. Academic satisfaction had a mean of 3.77 (SD = 0.95).

**Table 1:** Descriptive statistics for the study variables.

Variable	*n*	M	SD	Minimum	Maximum	Skewness	Kurtosis
Career adaptability	485	91.50	15.67	28	120	−0.48	0.43
Cognitive flexibility	485	76.11	11.21	37	100	0.01	−0.25
Self-esteem	485	31.67	5.30	13	40	−0.57	−0.02
General self-efficacy	485	31.81	5.48	13	40	−0.50	−0.15
Academic satisfaction	485	3.77	0.95	1	5	−0.85	0.58

Categorical variable	*n*	%
Career choice status		
Voluntary enrollment	362	75.1
Involuntary enrollment	120	24.9
Academic satisfaction		
Satisfied or completely satisfied	341	70.3
Undecided	92	19.0
Not satisfied or not satisfied at all	52	10.7

M, mean; SD, standard deviation. Minimum and maximum values indicate observed score ranges. Kurtosis values represent excess kurtosis. Academic satisfaction was assessed using a single item scored from 1 to 5. Career choice percentages are based on 482 valid responses; three undefined response codes were treated as missing. All other statistics are based on 485 participants.

Valid career choice responses were available for 482 participants. Of these, 362 (75.1%) reported choosing their current department willingly and 120 (24.9%) reported not doing so. Bivariate associations among the study variables were positive and statistically significant (all *p* < 0.01; Table 2). Self-esteem and cognitive flexibility were positively correlated in the full sample (Pearson’s *r* = 0.494, *p* < 0.001, *N* = 485), supporting the positive association proposed in H6.

**Table 2:** Intercorrelations and internal consistency estimates for the study variables.

Variable	1	2	3	4	5	6	α	ωₜ
1. Career adaptability	—						0.938	0.949
2. Cognitive flexibility	0.64**	—					0.890	0.905
3. Self-esteem	0.58**	0.49**	—				0.862	0.870
4. General self-efficacy	0.67**	0.63**	0.50**	—			0.880	0.881
5. Career choice status	0.20**	0.19**	0.16**	0.14**	—		—	—
6. Academic satisfaction	0.24**	0.18**	0.22**	0.15**	0.48**	—	—	—

Correlations among the four multi-item scale scores are Pearson correlations. Correlations between career choice status and these scale scores are point-biserial correlations. All correlations involving academic satisfaction are Spearman rank correlations. Career choice status was coded 0 = involuntary enrollment and 1 = voluntary enrollment. Correlations involving career choice status are based on 482 valid observations; all other correlations and reliability estimates are based on 485 observations. α = Cronbach’s alpha; ωₜ = model-based omega total. Reliability estimates refer to total scale scores. For multidimensional measures, omega total includes common variance across the correlated factors and does not imply unidimensionality. Internal consistency coefficients were not calculated for the two single-item measures.

**p < 0.01 (two-tailed).

### Internal consistency and measurement models

Internal consistency estimates for the four multi-item measures are presented in Table 2. Results of the separate confirmatory factor analyses, including model fit summaries and standardized item loadings, are presented in Supplementary Tables S1–S5. All items were retained, including Item 8 of the self-esteem scale, which showed a comparatively low standardized loading (0.259).

### Structural model and direct associations

The observed-variable path model was estimated using 482 complete records. Browne’s residual-based test did not indicate a statistically significant discrepancy between the observed and model-implied covariance structures, T_B = 7.296, df = 5, *p* = 0.200; SRMR was 0.020 (Table 3). The proportions of model-implied variance accounted for were 44.8% for general self-efficacy, 59.8% for career adaptability, 5.2% for career choice status, and 28.3% for academic satisfaction. These results describe the fitted linear observed-score model.

**Table 3:** Model fit and explained variance for the structural path model.

Model characteristic or outcome	Value
Model characteristics and fit	
Analytical sample size	482
Number of free parameters	16
Browne’s residual-based ADF test (T_B)	7.296
Degrees of freedom	5
*p* value	0.200
Standardized root mean square residual (SRMR)	0.020
Explained variance (R^2^)	
General self-efficacy	0.448
Career adaptability	0.598
Career choice status	0.052
Academic satisfaction	0.283

The observed-variable path model was estimated using unweighted least squares applied to the Pearson correlation matrix. Overall covariance fit was assessed using Browne’s (1984) residual-based asymptotically distribution-free (ADF) test, evaluated against a chi-square distribution with five degrees of freedom. SRMR summarizes standardized residual discrepancy. Career choice status and academic satisfaction were treated as numeric observed variables; the fit statistics therefore refer to a linear observed-score model. R^2^ values represent the proportion of variance explained in each endogenous variable.

Direct path estimates are presented in Table 4. Self-esteem was positively associated with general self-efficacy (β = 0.250, 95% CI [0.166, 0.332]), as was cognitive flexibility (β = 0.509, 95% CI [0.425, 0.586]), supporting H4 and H5. General self-efficacy (β = 0.325, 95% CI [0.237, 0.414]), self-esteem (β = 0.290, 95% CI [0.192, 0.388]), and cognitive flexibility (β = 0.310, 95% CI [0.209, 0.409]) were positively associated with career adaptability, supporting H1–H3.

**Table 4:** Direct path estimates and hypothesis decisions for the structural model.

Hypothesis	Structural path	B	SE(B)	β	95% CI for β	*p*	Decision
H1	General self-efficacy → Career adaptability	0.9191	0.1382	0.325	[0.237, 0.414]	<0.001	Supported
H2	Self-esteem → Career adaptability	0.8455	0.1439	0.290	[0.192, 0.388]	<0.001	Supported
H3	Cognitive flexibility → Career adaptability	0.4272	0.0658	0.310	[0.209, 0.409]	<0.001	Supported
H4	Self-esteem → General self-efficacy	0.2587	0.0438	0.250	[0.166, 0.332]	<0.001	Supported
H5	Cognitive flexibility → General self-efficacy	0.2479	0.0210	0.509	[0.425, 0.586]	<0.001	Supported
H7	General self-efficacy → Career choice status	0.0006	0.0064	0.007	[−0.162, 0.160]	0.931	Not supported
H8	Career adaptability → Career choice status	0.0063	0.0024	0.224	[0.075, 0.399]	0.008	Supported
H9	Career adaptability → Academic satisfaction	0.0092	0.0028	0.150	[0.061, 0.238]	0.001	Supported
H10	Career choice status → Academic satisfaction	1.0519	0.1082	0.478	[0.392, 0.556]	<0.001	Supported

*N* = 482. Parameters were estimated jointly using unweighted least squares. B = unstandardized coefficient in the original score units; SE(B) = bootstrap standard error of B; β = standardized coefficient based on model-implied variances. Confidence intervals are 95% percentile bootstrap intervals for β, based on 5,000 participant-level resamples. Reported *p* values are approximate two-sided Wald probabilities calculated from B and its bootstrap standard error. Career choice status was coded 0 = involuntary enrollment and 1 = voluntary enrollment; academic satisfaction was scored from 1 to 5. Arrows indicate the specified model direction and do not establish causality. H6 concerns the association between the two exogenous variables and is reported in Table 2 and the Findings text rather than as a directional path.

Career adaptability was positively associated with voluntary department choice (β = 0.224, 95% CI [0.075, 0.399]), supporting H8. The direct association between general self-efficacy and career choice status was not statistically distinguishable from zero (β = 0.007, 95% CI [−0.162, 0.160], *p* = 0.931); H7 was therefore not supported.

Academic satisfaction was positively associated with career adaptability (β = 0.150, 95% CI [0.061, 0.238]) and voluntary department choice (β = 0.478, 95% CI [0.392, 0.556]), supporting H9 and H10, respectively. All confidence intervals reported for the structural coefficients were 95% percentile bootstrap intervals.

The standardized structural model is presented in Figure 3.

**Figure 3:**
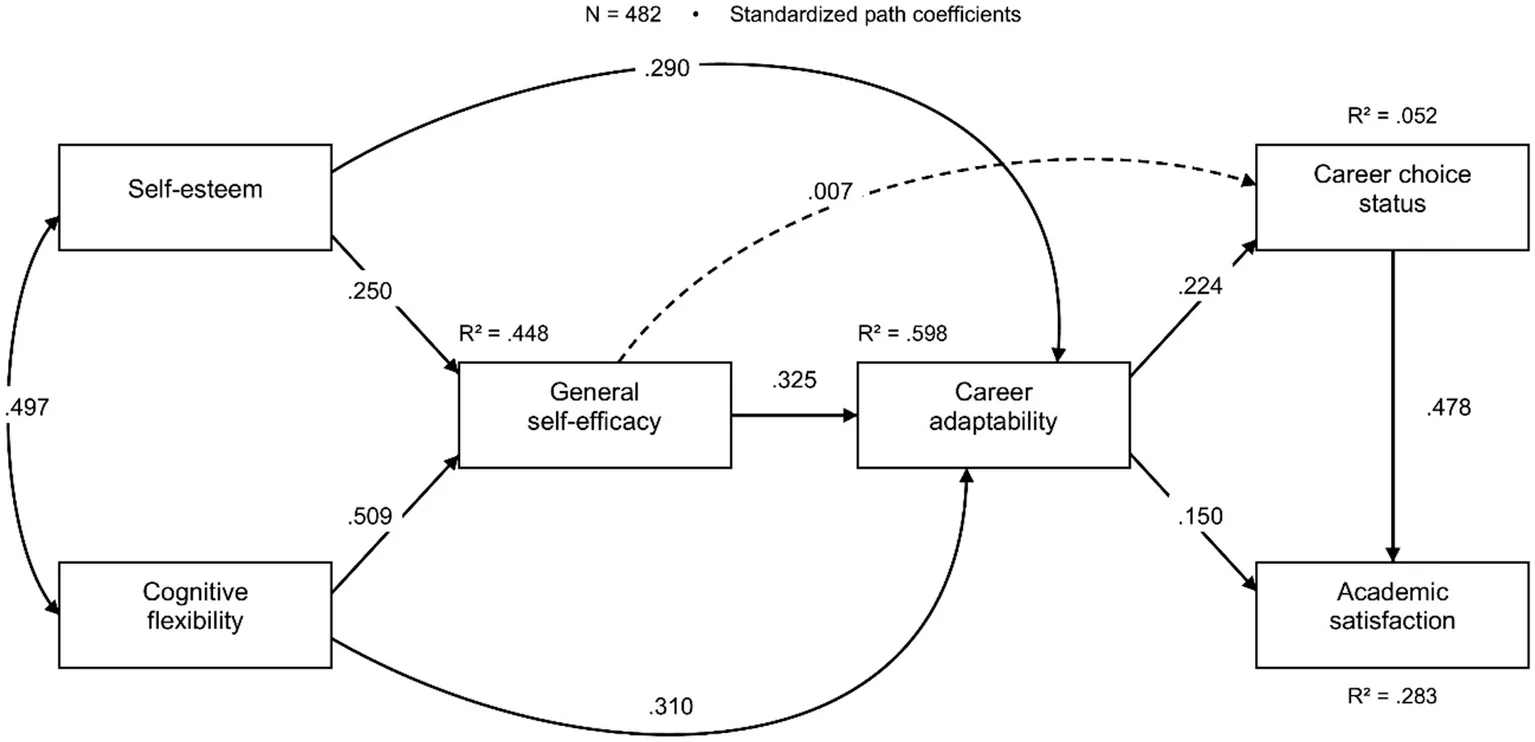
Standardized estimates for the structural path model. *N* = 482. Values beside single-headed arrows are standardized ULS path coefficients. R^2^ values indicate the proportion of variance explained in each endogenous variable. The dashed path from general self-efficacy to career choice status was not statistically significant (*p* = 0.931); all other directional paths had *p* < 0.01, based on approximate Wald tests using bootstrap standard errors. The value beside the double-headed arrow is the model-implied standardized covariance between self-esteem and cognitive flexibility (0.497), estimated using 482 complete records. The Pearson correlation reported in Table 2 is based on 485 participants. Residual terms are omitted for clarity. Career choice status was coded 0 = involuntary enrollment and 1 = voluntary enrollment; academic satisfaction was scored from 1 to 5. The diagram represents a linear observed-score model and does not establish causal effects.

### Specific indirect associations

The bootstrap confidence intervals excluded zero for all four specified indirect associations (Table 5). Self-esteem was indirectly associated with career adaptability through general self-efficacy (standardized indirect association = 0.081, 95% CI [0.051, 0.117]), supporting H11. The corresponding indirect association for cognitive flexibility was 0.166 (95% CI [0.115, 0.224]), supporting H12.

**Table 5:** Specific indirect associations and bootstrap confidence intervals.

Hypothesis	Indirect path	B	SE(B)	β	95% CI for β	Decision
H11	Self-esteem → General self-efficacy → Career adaptability	0.2377	0.0522	0.081	[0.051, 0.117]	Supported
H12	Cognitive flexibility → General self-efficacy → Career adaptability	0.2278	0.0403	0.166	[0.115, 0.224]	Supported
H13	General self-efficacy → Career adaptability → Career choice status	0.0058	0.0023	0.073	[0.023, 0.139]	Supported
H14	Career adaptability → Career choice status → Academic satisfaction	0.0066	0.0026	0.107	[0.036, 0.194]	Supported

*N* = 482. B = unstandardized indirect association, calculated as the product of the relevant unstandardized path coefficients; SE(B) = bootstrap standard error of B; β = standardized indirect association, calculated as the product of the corresponding standardized path coefficients. Confidence intervals are 95% percentile bootstrap intervals for β, based on 5,000 participant-level resamples with the full structural model re-estimated in each resample. An interval excluding zero was interpreted as evidence of a nonzero indirect association. Career choice status was coded 0 = involuntary enrollment and 1 = voluntary enrollment; academic satisfaction was scored from 1 to 5. The cross-sectional design does not establish temporal ordering or causal mediation. The nonsignificant direct association for H7 does not establish complete mediation for H13.

General self-efficacy was indirectly associated with career choice status through career adaptability (standardized indirect association = 0.073, 95% CI [0.023, 0.139]), supporting H13. The nonsignificant direct association did not establish that this relationship operated exclusively through career adaptability.

Career adaptability was indirectly associated with academic satisfaction through career choice status (standardized indirect association = 0.107, 95% CI [0.036, 0.194]), supporting H14. These findings were consistent with the hypothesized indirect associations but did not establish temporal ordering or causal mediation.

### Sensitivity analyses

The categorical outcome models showed the same direction and pattern of statistical evidence for the corresponding direct associations. In the binary logistic model, career adaptability was positively associated with voluntary department choice (OR = 1.028, 95% CI [1.008, 1.048], *p* = 0.005), whereas general self-efficacy was not significantly associated with it (OR = 1.008, 95% CI [0.957, 1.063], *p* = 0.753). These odds ratios correspond to a one-point increase in the respective scale total scores.

In the ordinal logistic model, a one-point increase in career adaptability was associated with higher odds of reporting greater academic satisfaction (OR = 1.021, 95% CI [1.009, 1.032], *p* < 0.001). Participants who reported choosing their department willingly also had higher odds of reporting greater academic satisfaction than those who did not (OR = 9.780, 95% CI [6.232, 15.346], *p* < 0.001).

These supplementary analyses supported the direction and statistical evidence for the corresponding direct associations under alternative outcome specifications. They did not independently test the full system of indirect associations.

## Discussion

The findings of this study are broadly consistent with the theoretical propositions of career construction theory (CCT; Savickas, 2002, 2005, 2013) and provide evidence relevant to the career construction model of adaptation (CCMA) among university students in Türkiye. Eight of the nine directional paths were statistically significant, as was the positive correlation between self-esteem and cognitive flexibility. Bootstrap confidence intervals also supported all four specified indirect associations. Together, these findings help clarify how personal resources and career adaptability are associated with career choice status and academic satisfaction, while the cross-sectional design precludes conclusions about temporal ordering or causal mechanisms.

### Synthesis of adaptability precursors and adaptational readiness

A pivotal contribution of this study lies in its joint evaluation of the psychological and socio-cognitive characteristics relevant to university students’ adaptive readiness. The findings indicated that self-esteem, cognitive flexibility, and general self-efficacy were positively associated with career adaptability within the same structural model. General self-efficacy had the numerically largest standardized direct coefficient for career adaptability (β = 0.325; H1), although differences between the three coefficients were not formally tested. From a theoretical standpoint, a stronger belief in one’s ability to manage challenges may support the mobilization of career-related coping resources. This interpretation is consistent with research linking efficacy beliefs to career adaptability (Hirschi, 2009; Marcionetti and Rossier, 2021; Xu, 2020), including evidence from Türkiye (Karacan-Özdemir, 2016; Öncel, 2014). Related research has also highlighted the relevance of both general self-efficacy and career adaptability to perceived employability (Atitsogbe et al., 2019). Together, these findings suggest that general efficacy beliefs are relevant to understanding career adaptability, while remaining conceptually distinct from career-specific adaptability resources.

Furthermore, the significant positive correlation between cognitive flexibility and self-esteem (H6) indicates that flexible thinking and positive self-evaluation are related aspects of students’ psychological functioning. Students reporting greater self-worth also tended to report greater cognitive flexibility. Their positive associations with career adaptability in the structural model suggest that both characteristics are relevant when considered alongside general self-efficacy. This pattern supports examining these personal characteristics together within the adaptive readiness component of the CCMA. However, the correlation does not establish that self-esteem and cognitive flexibility reinforce one another over time, and the cross-sectional findings do not determine the direction of their relationship.

### Direct and indirect associations of self-esteem and cognitive flexibility

Expanding upon the individual pathways, the structural findings indicated that self-esteem was positively associated with career adaptability both directly (β = 0.290; H2) and indirectly through general self-efficacy (standardized indirect association = 0.081; H11). From a theoretical standpoint, this pattern suggests that students’ sense of self-worth may be relevant to career adaptability both in its own right and through its association with broader beliefs about their ability to manage challenges (H4). Students with more positive self-evaluations may perceive themselves as better equipped to address vocational demands, a possible interpretation of the observed associations rather than an established developmental mechanism. This pattern is broadly consistent with vocational research linking self-esteem and related personal resources to career development (Cai et al., 2015; Hui et al., 2018; Lee et al., 2021; Marcionetti and Rossier, 2021), including findings from Türkiye (Ataç et al., 2018; Boyacı, 2019; Öncel, 2014; Öztemel and Yıldız-Akyol, 2021).

Similarly, cognitive flexibility was positively associated with career adaptability directly (β = 0.310; H3) and indirectly through general self-efficacy (standardized indirect association = 0.166; H12). Its positive association with general self-efficacy (H5) is consistent with the possibility that students who can consider alternative perspectives also feel more capable of managing difficult situations. From a theoretical perspective, flexible thinking may help students consider diverse strategies when confronting career obstacles, making it relevant to the development and use of adaptability resources. This interpretation aligns with research examining cognitive flexibility in relation to career adaptability (Chong and Leong, 2017; Yıldız-Akyol and Boyacı, 2020). Nevertheless, the present findings do not establish that cognitive flexibility strengthens efficacy beliefs over time or that changes in these beliefs subsequently produce greater career adaptability.

### Joint contributions of adaptive readiness characteristics

A central contribution of this study is the simultaneous examination of self-esteem, cognitive flexibility, and general self-efficacy within a single structural model. Together, these three personal characteristics accounted for 59.8% of the model-implied variance in career adaptability (*R*^2^ = 0.598). Each retained a positive direct association with career adaptability when considered alongside the others. This pattern suggests that cognitive flexibility, positive self-evaluation, and generalized efficacy beliefs offer complementary perspectives on adaptive readiness. Their joint examination therefore contributes to understanding how distinct but related personal characteristics are associated with adaptability resources, without implying that statistical interactions or synergistic effects were tested.

In the contemporary labor market, characterized by economic and technological uncertainty, these findings highlight the relevance of personal resources to university students’ career preparation. Alongside technical and academic skills, self-esteem, general self-efficacy, and cognitive flexibility may be useful areas for university career counselors to consider when exploring students’ perceived strengths and difficulties. However, the present study does not establish that these resources compensate for limited institutional support or that interventions targeting them would improve career adaptability. Such possibilities require direct investigation through longitudinal and intervention research.

### Career adaptability and career choice status

Moving beyond the personal precursors, the findings provide insight into the associations between career adaptability and career choice status within the CCMA framework. Career adaptability was positively associated with career choice status (β = 0.224; H8), defined here as students’ reports of having chosen their current academic department willingly. General self-efficacy showed a significant indirect association with career choice status through career adaptability (standardized indirect association = 0.073, 95% CI [0.023, 0.139]; H13), whereas its direct association was not statistically distinguishable from zero (β = 0.007, 95% CI [−0.162, 0.160]; H7). This pattern supports the proposed indirect association but does not establish full mediation or demonstrate that general self-efficacy relates to career choice status exclusively through career adaptability.

According to Career Construction Theory, adaptability resources help individuals address vocational tasks and transitions, providing a theoretical link between adaptive readiness and adapting responses (Savickas, 2005, 2013). The present pattern is consistent with the possibility that career-specific resources are more closely related to students’ reported willingness in department choice than broad beliefs about their capacity to manage challenges. However, the nonsignificant direct association should not be interpreted as evidence that general self-efficacy is irrelevant to educational choices. Its association may overlap with career adaptability, while the binary, single-item indicator may not capture other aspects of decision-making to which efficacy beliefs are relevant. Moreover, department choice preceded the assessment of current self-efficacy and career adaptability. Experiences within the chosen department may therefore have shaped these resources, and current satisfaction may influence recollections of the original choice. These alternative explanations limit conclusions about the direction and timing of the proposed relationships.

The positive association between career adaptability and willing department choice can be considered alongside international research on adaptability and students’ relationships with their chosen fields. Wessel et al. (2008) found that individual adaptability was positively related to affective commitment to the academic major and negatively related to the reported probability of changing majors. Although their measure of individual adaptability differs from the career adaptability construct assessed here, their findings suggest that adaptive capacities are relevant to how students experience and maintain their educational choices. Similarly, Perera and McIlveen (2017) found that students in the “adaptive ready” profile reported both higher career adaptability and greater satisfaction with their occupational choice than those in the “ordinary” and “rigid” profiles. Wilkins-Yel et al. (2018) further reported an indirect association between career adaptability and intended academic persistence through academic satisfaction. Together, these studies connect adaptability with commitment, satisfaction, and persistence-related aspects of educational and career choice. The present study complements this literature by examining a different aspect of the choice experience: whether students reported having selected their current department willingly.

The Turkish evidence provides a particularly relevant comparison for this finding. In their mixed-methods study of preservice teachers, Erus and Zeren (2017) found that interviewees with higher career adaptability scores described choosing their profession consciously and willingly, whereas most of those with lower scores attributed their choices to university entrance examination scores. This qualitative contrast is consistent with the positive association between career adaptability and willing department choice observed in the present study, although their focus was on choosing the teaching profession rather than willingness in department choice across different fields. Their findings also illustrate why the Turkish university placement context matters to the interpretation of our results: students may describe their choices in terms of personal intentions as well as examination-related opportunities and constraints. The present data do not directly measure these constraints, but they allow reported willingness in department choice to be considered alongside personal resources and career adaptability.

Similarly, Yıldız-Akyol and Boyacı (2020) found that Turkish university students who reported selecting their majors voluntarily had higher career future scores than those who reported choosing unwillingly. Career adaptability is a subdimension of the Career Futures Inventory used in their study, alongside career optimism and perceived knowledge of the labor market. Their findings therefore provide a relevant comparison for the present association between career adaptability and willing department choice, although the reported difference in overall career future scores does not isolate the contribution of the adaptability subdimension. Building on this evidence, the present study examines career adaptability specifically and places career choice status within a model that also includes self-esteem, cognitive flexibility, general self-efficacy, and academic satisfaction. Its contribution is to examine how reported willingness in department choice relates to adaptability resources and educational satisfaction within the same framework. This offers evidence relevant to the CCMA in Turkish higher education, while leaving open whether similar relationships would emerge across institutions and cultural settings.

### Career adaptability, career choice status, and academic satisfaction

The findings indicated that career adaptability was positively associated with academic satisfaction both directly (β = 0.150; H9) and indirectly through career choice status (standardized indirect association = 0.107, 95% CI [0.036, 0.194]; H14). Students who reported choosing their departments willingly also reported greater academic satisfaction, accounting for career adaptability (β = 0.478; H10). From a theoretical standpoint, this pattern suggests that adaptability resources and the perceived willingness of an earlier educational choice are both relevant to students’ evaluations of their current academic experience. The positive association between career adaptability and academic satisfaction is consistent with research examining adaptability in relation to educational satisfaction (Celik and Storme, 2018; Norris, 2016; Xu, 2020; Wilkins-Yel et al., 2018). Studies by Duffy et al. (2015) and Nilforooshan (2020) further suggest that this relationship may involve intervening variables, including work volition, career decision-making self-efficacy, and career engagement. The present study complements these accounts by examining reported willingness in department choice as a distinct potential link between career adaptability and academic satisfaction. However, this indirect association does not establish that current adaptability shaped the earlier choice or that willingness in choice subsequently caused greater satisfaction.

Career adaptability and career choice status together accounted for 28.3% of the model-implied variance in academic satisfaction (R^2^ = 0.283). This finding highlights the relevance of considering students’ adaptability resources alongside their reported willingness in department choice when examining satisfaction with their academic experience. In terms of the CCMA, the observed pattern is consistent with examining adaptability resources in relation to both a choice-related indicator and an adaptation outcome. The remaining direct association between career adaptability and academic satisfaction also indicates that the modeled indirect association through career choice status does not fully account for their relationship. Rather than validating every stage of the adaptation process, these results provide evidence for a specific set of associations within the proposed framework. Other personal and contextual factors not included in the model may also contribute to academic satisfaction.

The model fit results provided additional evidence that the proposed relationships were compatible with the observed covariance structure. The residual-based test did not indicate a statistically significant discrepancy between the observed and model-implied covariances (T_B = 7.296, df = 5, *p* = 0.200), and the SRMR was 0.020. These findings support the model as a plausible representation of the associations in this sample. However, satisfactory fit does not establish that the proposed model is uniquely correct, demonstrate the direction of the relationships, or validate the CCMA across cultural settings. The findings should therefore be interpreted alongside research examining the adaptation framework in other samples and with different operationalizations (Lee et al., 2021; Nilforooshan, 2020; Öztemel and Yıldız-Akyol, 2021; Šverko and Babarović, 2019).

Taken together, the findings suggest that self-esteem, cognitive flexibility, and general self-efficacy are relevant to under

standing career adaptability, while career adaptability and willing department choice are associated with academic satisfaction. The study’s contribution lies in examining these relationships within an integrated model that distinguishes broad personal characteristics, career-specific adaptability resources, and students’ evaluations of their educational choices and experiences. The significant indirect associations are consistent with the proposed CCMA-informed pathways, but they do not establish a developmental sequence. Within the Turkish higher education context, the results highlight reported willingness in department choice as a relevant link to consider when investigating the relationship between career adaptability and academic satisfaction.

### Implications for practice

The findings suggest several potential directions for university career counseling. Self-esteem, cognitive flexibility, and general self-efficacy each showed positive associations with career adaptability, making them relevant areas to consider when exploring students’ career preparation and support needs. Counselors could consider psychoeducational activities addressing self-evaluations, alternative ways of approaching career difficulties, and beliefs about managing challenges. Cognitive-behavioral exercises and narrative activities may offer ways to explore these themes, provided their selection is guided by students’ needs and relevant intervention evidence. Group counseling could similarly provide opportunities to explore career concern, control, curiosity, and confidence through planning, decision-making, exploration, and problem-solving activities. The present findings identify potentially relevant targets for such work, but do not establish the effectiveness of particular counseling approaches.

For university career centers, the positive association between willing department choice and academic satisfaction highlights the value of discussing students’ interests, expectations, and experiences of their educational decisions. Department-specific information about occupational opportunities and structured alumni mentoring could provide settings for exploring how current studies relate to possible career pathways. For students who report choosing their departments unwillingly, these activities could create opportunities to reconsider available options, identify meaningful aspects of their programs, or explore alternative pathways without assuming that changing departments is necessarily the appropriate solution. These are possible applications to be evaluated, rather than interventions whose benefits were demonstrated by the present study.

For educators and academic advisers, the findings suggest that career preparation may usefully be considered alongside academic learning. Interactive learning activities, opportunities to consider alternative solutions, and supportive communication could provide settings in which students reflect on their capabilities and future goals. Collaboration with career counselors may help connect these educational activities with students’ individual career concerns. Given the association of academic satisfaction with both career adaptability and willing department choice, advisers could also include questions about career expectations and experiences of department choice in discussions of students’ academic progress.

Future evaluations should examine whether these proposed activities produce changes in the intended personal resources, career adaptability, and academic satisfaction. Longitudinal and intervention research would help determine which components are useful, for whom, and under what conditions. Until such evidence is available, the present results should guide the identification of relevant support themes rather than be treated as proof that a particular program will improve students’ outcomes.

### Limitations and future research directions

Several limitations should be considered when interpreting these findings. First, the cross-sectional design prevents conclusions about temporal ordering and causality. Although the directional paths were guided by Career Construction Theory, alternative directions of association remain possible. In particular, department choice preceded the assessment of current personal resources and career adaptability. Experiences within the chosen department may have shaped these resources, while current academic satisfaction may influence how students recall their earlier choices. Future research should use longitudinal designs that assess personal resources and adaptability before educational decisions and follow students’ subsequent experiences. Qualitative research could complement these designs by examining how students interpret their choices and career development over time.

Second, career choice status and academic satisfaction were assessed using single-item indicators. These items provided focused information about willingness in department choice and satisfaction with the current department, but their limited content coverage restricts the interpretation of the corresponding CCMA components. Reported willingness does not capture the full range of adapting responses, such as career exploration, planning, and decision-making behaviors. Similarly, satisfaction with the current department does not encompass all aspects of academic adjustment or university life. Internal consistency cannot be estimated for these single-item indicators, and measurement error remains a concern. Future studies should incorporate multi-item measures of adapting responses and academic satisfaction to examine whether the proposed relationships hold across more comprehensive operationalizations.

Third, all measures were collected from the same respondents on one occasion, creating the possibility that shared response tendencies and common method variance contributed to the observed associations. No dedicated statistical assessment of common method bias was conducted; the reliability estimates and measurement-model results should not be interpreted as ruling out this possibility. Future research could incorporate temporal separation, multiple information sources where appropriate, and designs that allow substantive relationships to be distinguished more clearly from method effects.

Fourth, the structural model used observed scale totals and treated the binary career choice indicator and ordinal academic satisfaction item as numeric scores within a linear ULS framework. This approach does not explicitly model categorical response thresholds or correct structural coefficients for measurement error. Binary and ordinal logistic sensitivity analyses supported the direction and pattern of statistical evidence for the corresponding direct associations, but they did not independently test the full system of indirect associations. Future studies could compare the present results with models that explicitly accommodate categorical outcomes and measurement error. In addition, participant-level bootstrapping treated observations as independent and did not account for possible classroom clustering.

Finally, participants were recruited through convenience sampling at a single university in Türkiye. The findings therefore cannot be assumed to represent all Turkish university students or students in other educational systems. Although the university placement context informed the interpretation, institutional constraints and cultural influences were not directly measured. Multi-institutional and cross-cultural research is needed to examine the generalizability of the relationships and the conditions under which they may differ.

## Data Availability

The original contributions presented in the study are included in the article/Supplementary material, further inquiries can be directed to the corresponding author.
