# Supplementary material for: Career construction model of adaptation: an empirical study among university students in Türkiye
Source: Front Psychol. 2026 Oct 5;17:1865636. doi: 10.3389/fpsyg.2026.1865636 (PMC13677684; doi:10.3389/fpsyg.2026.1865636)
Supplement: Supplementary file 1 [file Supplementary_file_1.pdf]

Supplementary Material S1

Confirmatory Factor Analysis Results

Separate confirmatory factor analyses were conducted for the four multi-item measures using all 485 participants. Models were estimated in semopy, version 2.3.11, using unweighted least squares applied to Pearson item correlation matrices. Item responses were treated as numeric scores; these analyses were not threshold-based ordinal factor models.

The Career Adapt-Abilities Scale specified four correlated factors, the Cognitive Flexibility Inventory specified two correlated factors, and the Rosenberg Self-Esteem Scale and General Self-Efficacy Scale each specified one factor. No cross-loadings or correlated residuals were added. Internal consistency estimates for the total scores are reported in Table 2 of the main manuscript.

Table S1

Summary of the Separate Measurement Models

**Measure**	**Factor structure**	**df**	**SRMR**	**Standardized loading range**
Career Adapt-Abilities Scale	Four correlated factors	246	.048	.465–.823
Cognitive Flexibility Inventory	Two correlated factors	169	.072	.387–.794
Rosenberg Self-Esteem Scale	One factor	35	.070	.259–.769
General Self-Efficacy Scale	One factor	35	.050	.563–.728

Note. N = 485. All four models converged, with positive estimated residual variances and positive-definite factor covariance matrices. SRMR = standardized root mean square residual. These results describe separate scale-level measurement models, not the structural path model. No calibrated chi-square test was computed for these measurement models; consequently, chi-square-based CFI, TLI, and RMSEA values are not reported. The residual-based test reported for the structural model should not be attributed to these measurement models.

Table S2

Standardized Factor Loadings for the Career Adapt-Abilities Scale

**Item**	**Factor**	**Standardized loading**
1	Concern	.622

2	Concern	.678
3	Concern	.823
4	Concern	.747
5	Concern	.776
6	Concern	.799
7	Control	.614
8	Control	.612
9	Control	.728
10	Control	.465
11	Control	.763
12	Control	.757
13	Curiosity	.624
14	Curiosity	.802
15	Curiosity	.732
16	Curiosity	.721
17	Curiosity	.624
18	Curiosity	.741
19	Confidence	.659
20	Confidence	.629
21	Confidence	.702
22	Confidence	.719
23	Confidence	.757
24	Confidenc	.740

e

Note. Item numbers correspond to the numbered item variables in the analyzed dataset (kuyo_1–kuyo_24).

Table S3

Standardized Factor Loadings for the Cognitive Flexibility Inventory

Item	Factor	Standardized loading
1	Alternatives	.578
2	Control	.387
3	Alternatives	.575
4	Control	.530
5	Alternatives	.602
6	Alternatives	.653
7	Control	.617
8	Alternatives	.481
9	Control	.389
10	Alternatives	.449
11	Control	.629
12	Alternatives	.580
13	Alternatives	.733
14	Alternatives	.740
15	Control	.792

16	Alternatives	.635
17	Control	.664
18	Alternatives	.794
19	Alternatives	.791
20	Alternatives	.782

Note. Item numbers correspond to the numbered item variables in the analyzed dataset (bee_1–bee_20). Loadings refer to the scored item variables.

Table S4

Standardized Factor Loadings for the Rosenberg Self-Esteem Scale

Item	Factor	Standardized loading
1	Self-esteem	.660
2	Self-esteem	.599
3	Self-esteem	.582
4	Self-esteem	.544
5	Self-esteem	.682
6	Self-esteem	.742
7	Self-esteem	.737
8	Self-esteem	.259
9	Self-esteem	.769

10	Self-esteem	.766

Note. Item numbers correspond to the numbered item variables in the analyzed dataset (rbso_1–rbso_10). Loadings refer to the scored item variables. Item 8 showed a comparatively low loading (.259). All ten items were retained; no item was removed to improve model fit.

Table S5

Standardized Factor Loadings for the General Self-Efficacy Scale

Item	Factor	Standardized loading
1	General self-efficacy	.572
2	General self-efficacy	.626
3	General self-efficacy	.632
4	General self-efficacy	.728
5	General self-efficacy	.683
6	General self-efficacy	.634
7	General self-efficacy	.563
8	General self-efficacy	.702
9	General self-efficacy	.690
10	General self-efficacy	.679

Note. Item numbers correspond to the numbered item variables in the analyzed dataset (goyo_1–goyo_10).
