# Supplementary material for: Career construction model of adaptation: an empirical study among university students in Türkiye
Source: Front Psychol. 2026 Oct 5;17:1865636. doi: 10.3389/fpsyg.2026.1865636 (PMC13677684; doi:10.3389/fpsyg.2026.1865636)
Supplement: Supplementary file 2 [file Supplementary_file_2.pdf]

Supplementary Material S2

Study Measures and Scoring Instructions

This supplement presents the Turkish item wording and response options of the four multi-item measures and the two personal-information items used in the structural model. English descriptions explain the scoring procedures; the Turkish wording is retained to document the study instruments.

The structural model used total scores for the four multi-item measures. Higher scores indicate higher levels of the corresponding construct. Item numbers correspond to those used in Supplementary Material S1.

1. Career Adapt-Abilities Scale

Kariyer Uyum Yetenekleri Ölçeği

Sources: Savickas and Porfeli (2012); Turkish adaptation by Buyukgoze-Kavas (2014).

Instructions in Turkish

İnsanlar kariyerlerini şekillendirirken güçlü oldukları farklı yanlarını kullanırlar. Hiç kimse her konuda iyi olamaz. Her birimizin diğerlerinden daha güçlü olduğu bazı yanları vardır. Aşağıda belirtilen her bir konuda kendinizi ne kadar güçlü/iyi geliştirdiğinizi belirtiniz.

Response options

1 = Hiç Güçlü Değil
2 = Az Güçlü
3 = Biraz Güçlü
4 = Oldukça Güçlü
5 = Çok Güçlü

Items

1. Geleceğimin nasıl olacağı hakkında düşünme
2. Bugünkü seçimlerimin geleceğimi şekillendireceğinin farkında olma
3. Gelecek için hazırlanma
4. Eğitim ve meslekle ilgili yapmam gereken seçimlerin farkına varma
5. Hedeflerime nasıl ulaşacağımı planlama
6. Kariyerimle ilgilenme
7. Moralimi, neşemi sürdürebilme
8. Kendi kendime karar verme

9. Davranışlarımın sorumluluğunu alma
10. Kendi doğrularıma bağlı kalma
11. Kendime güvenme
12. Kendim için doğru olanı yapma
13. Çevremi keşfetme
14. Bir birey olarak gelişimim için fırsatlar arama
15. Herhangi bir seçim yapmadan önce seçenekleri inceleme
16. İşlerin farklı yapılış yollarını gözlemleme
17. Aklımdaki soruları derinlemesine irdeleme
18. Yeni olanaklar hakkında meraklı olma
19. Görevleri etkili bir şekilde yerine getirme
20. İşleri iyi yapmaya özen gösterme
21. Yeni beceriler kazanma
22. Yeteneklerim doğrultusunda çalışma
23. Engellerin üstesinden gelme
24. Problemleri çözme

Scoring

All 24 items are positively scored. The total score is the sum of the item scores and ranges from 24 to 120. No items are reverse-scored.

The four subscales comprise Concern (Items 1–6), Control (Items 7–12), Curiosity (Items 13–18), and Confidence (Items 19–24). Each subscale sum ranges from 6 to 30. The total score was used in the structural model.

2. Cognitive Flexibility Inventory

Bilişsel Esneklik Envanteri

Sources: Dennis and Vander Wal (2010); Turkish adaptation by Sapmaz and Doğan (2013).

Response options

1 = Hiç Uygun Değil
2 = Uygun Değil
3 = Biraz Uygun

4 = Uygun
5 = Tamamen Uygun

Items

1. Olay ve durumları değerlendirmede iyiyimdir.
2. Zor durumlarla karşı karşıya kaldığımda karar vermekte güçlük çekerim.
3. Bir konuda karar vermeden önce birçok seçeneği göz önünde bulundururum.
4. Zor durumlarla karşılaştığım zaman, kontrolümü kaybediyor gibi hissederim.
5. Zor durumlara pek çok farklı açılardan bakmak hoşuma gider.
6. Bir davranışın nedenleri konusunda karara varmadan önce ek bilgiler ararım.
7. Zor durumlarla karşılaştığımda o kadar stresli olurum ki, sorunu nasıl çözeceğimi düşünemem.
8. Olayları karşımdaki kişilerin bakış açılarından da görmeye çalışırım.
9. Zor durumlarla baş edebilmek için önümde farklı birçok seçeneğin olması benim için sıkıntı vericidir.
10. Kendimi diğer insanların yerine koyarak onları anlamada iyiyimdir.
11. Zor durumlarla karşılaştığımda tam olarak ne yapacağımı bilemem.
12. Zor durumlara değişik açılardan bakmak önemlidir.
13. Bir zorlukla karşılaştığımda nasıl davranacağımla ilgili olarak pek çok seçeneği göz önünde bulundururum.
14. Herhangi bir duruma çoğu kez farklı açılardan bakarım.
15. Yaşamda karşılaştığım güçlüklerle baş etmede yetenekliyim.
16. Davranışların nedenlerini anlamaya çalışırken bütün olası gerçekleri ve bilgileri göz önünde bulundururum.
17. Zor durumlarda bir şeyleri değiştirme konusunda kendimi güçsüz hissederim.
18. Zor bir durumla karşılaştığımda durur ve çözüm için çeşitli yollar düşünürüm.
19. Bir sorunla karşılaştığım zaman sorunu çözebilmek için birden fazla yol olduğunu düşünürüm.
20. Zor durumlarla ilgili harekete geçmeden önce pek çok seçeneği değerlendiririm.

Scoring

Items 2, 4, 7, 9, 11, and 17 are reverse-scored using 6 minus the original response: 1 becomes 5, 2 becomes 4, 3 remains 3, 4 becomes 2, and 5 becomes 1. After reverse-scoring, all 20 item scores are summed. Total scores range from 20 to 100.

The Control subscale comprises Items 2, 4, 7, 9, 11, 15, and 17. The Alternatives subscale comprises Items 1, 3, 5, 6, 8, 10, 12, 13, 14, 16, 18, 19, and 20. The total score was used in the structural model.

3. Rosenberg Self-Esteem Scale

Rosenberg Benlik Saygısı Ölçeği

Sources: Rosenberg (1965); Turkish adaptation by Çuhadaroğlu (1986).

Instructions in Turkish

Aşağıda kendinize ilişkin duygu ve düşüncelerinizi yansıtabileceğiniz bazı maddeler yer almaktadır. Lütfen her bir maddeyi dikkatlice okuyun ve kendiniz hakkında nasıl hissettiğinizi uygun seçeneği işaretleyerek belirtiniz.

Response options

1 = Hiç Katılmıyorum
2 = Katılmıyorum
3 = Katılıyorum
4 = Tamamen Katılıyorum

Items

1. Kendimi en az diğer insanlar kadar değerli buluyorum.
2. Bazı olumlu özelliklerimin olduğunu düşünüyorum.
3. Genelde kendimi başarısız bir kişi olarak görme eğilimindeyim.
4. Ben de diğer insanların çoğunun yapabileceği kadar bir şeyler yapabilirim.
5. Kendimde gurur duyacak fazla bir şey bulamıyorum.
6. Kendime karşı olumlu bir tutum içerisindeyim.
7. Genel olarak kendimden memnunum.
8. Kendime karşı daha fazla saygı duyabilmeyi isterdim.
9. Bazen kesinlikle kendimin bir işe yaramadığını düşünüyorum.
10. Bazen kendimin hiç de yeterli bir insan olmadığımı düşünüyorum.

Scoring

The study used the 10-item self-esteem measure. Items 3, 5, 8, 9, and 10 are reverse-scored using 5 minus the original response: 1 becomes 4, 2 becomes 3, 3 becomes 2,

and 4 becomes 1. After reverse-scoring, the 10 item scores are summed, yielding a total score from 10 to 40. Higher scores indicate greater self-esteem. This describes the scoring used in the present study.

4. General Self-Efficacy Scale

Genel Öz Yeterlik Ölçeği

Sources: Schwarzer and Jerusalem (1995); Turkish adaptation by Aypay (2010).

Instructions in Turkish

Bu ölçek, bireylerin stresli yaşantılarla başa çıkabilme ve bunlara uyum sağlayabilme becerilerine yönelik algılarını belirlemek amacıyla geliştirilmiştir. Aşağıda bazı düşünceleri içeren ifadeler yer almaktadır. Bu ifadelere katılma derecenizi “Tamamen yanlış”, “Biraz doğru”, “Orta düzeyde doğru”, “Tamamen doğru” seçeneklerinden size en uygun olanı işaretleyerek göstermeniz beklenmektedir. Lütfen her bir ifadede belirtilen düşüncenin size ne kadar uyduğunu düşününüz. Her bir ifadeye katılma derecenizi kendinize en uygun gelen seçeneğin altındaki kutucuğu işaretleyerek gösteriniz. Lütfen hiçbir maddeyi yanıtsız bırakmayınız. Değerli katkılarız için teşekkür ederim.

Response options

1 = Tamamen Yanlış
2 = Biraz Doğru
3 = Orta Düzeyde Doğru
4 = Tamamen Doğru

Items

1. Yeterince çaba harcarsam, zor sorunları çözmenin bir yolunu daima bulabilirim.
2. Bana karşı çıkıldığında, istediğimi elde etmemi sağlayacak bir yol ve yöntem bulabilirim.
3. Amaçlarıma bağlı kalmak ve bunları gerçekleştirmek benim için kolaydır.
4. Beklenmedik olaylarla etkili bir biçimde başa çıkabileceğime inanıyorum.
5. Yeteneklerim sayesinde beklenmedik durumlarla nasıl baş edebileceğimi biliyorum.
6. Gerekli çabayı gösterirsem, birçok sorunu çözebilirim.
7. Baş etme gücüme güvendiğim için zorluklarla karşılaştığımda soğukkanlılığımı koruyabilirim.
8. Bir sorunla karşılaştığımda, genellikle birkaç çözüm yolu bulabilirim.

9. Başım dertte olduğunda, genellikle bir çözüm düşünebilirim.
10. Önüme çıkan zorluk ne olursa olsun, üstesinden gelebilirim.

Scoring

All 10 items are positively scored. Item scores are summed to obtain a total score ranging from 10 to 40. No items are reverse-scored. Higher scores indicate greater general self-efficacy.

5. Personal Information Form

Kişisel Bilgi Formu

The personal information form included demographic questions and the following two items used in the structural model.

Career choice status

Turkish item wording

Bölümünüzü isteyerek mi tercih ettiniz?

Response options

Evet
Hayır

English explanation and analytical coding

The item asks whether participants chose their current academic department willingly. Valid responses were coded as 1 = yes and 0 = no for analysis. Three responses containing codes outside the defined categories were treated as missing.

Academic satisfaction

Turkish item wording

Bölümünüzden memnuniyet durumunuz:

Response options in the order displayed on the form

Tamamen Memnunum
Memnunum
Kararsızım
Memnun değilim
Hiç Memnun Değilim

English explanation and analytical coding

The item assesses satisfaction with the current academic department. Responses were scored as follows:

1 = Hiç Memnun Değilim
2 = Memnun değilim
3 = Kararsızım
4 = Memnunum
5 = Tamamen Memnunum

Higher scores indicate greater satisfaction with the current department. The numerical scoring increases with satisfaction, although the response options were displayed in descending order on the questionnaire.

The two items are separate indicators and are not combined into a total score. Internal consistency coefficients are not applicable to either single-item measure.
